# The mRNA expression of SATB1 and SATB2 in human breast cancer

**DOI:** 10.1186/1475-2867-9-18

**Published:** 2009-07-30

**Authors:** Neill Patani, Wen Jiang, Robert Mansel, Robert Newbold, Kefah Mokbel

**Affiliations:** 1 Department of Breast Surgery, St. George's University of London, London, UK; 2 The London Breast Institute, The Princess Grace Hospital, London, UK; 3 Metastasis and Angiogenesis Research Group, University Department of Surgery, Cardiff University, Cardiff, UK; 4 The Brunel Institute of Cancer Genetics and Pharmacogenomics, London, UK

## Abstract

**Background:**

SATB1 is a nuclear protein that has been recently reported to be a 'genome organizer' which delineates specific epigenetic modifications at target gene loci, directly up-regulating metastasis-associated genes while down-regulating tumor-suppressor genes. In this study, the level of mRNA expression of SATB1 and SATB2 were assessed in normal and malignant breast tissue in a cohort of women with breast cancer and correlated to conventional clinico-pathological parameters.

**Materials and methods:**

Breast cancer tissues (n = 115) and normal background tissues (n = 31) were collected immediately after excision during surgery. Following RNA extraction, reverse transcription was carried out and transcript levels were determined using real-time quantitative PCR and normalized against β-actin expression. Transcript levels within the breast cancer specimens were compared to the normal background tissues and analyzed against TNM stage, nodal involvement, tumour grade and clinical outcome over a 10 year follow-up period.

**Results:**

The levels of SATB1 were higher in malignant compared with normal breast tissue (p = 0.0167). SATB1 expression increased with increasing TNM stage (TNM1 vs. TNM2 p = 0.0264), increasing tumour grade (grade1 vs. grade 3 p = 0.017; grade 2 vs. grade 3 p = 0.0437; grade 1 vs. grade 2&3 p = 0.021) and Nottingham Prognostic Index (NPI) (NPI-1 vs. NPI-3 p = 0.0614; NPI-2 vs. NPI-3 p = 0.0495). Transcript levels were associated with oestrogen receptor (ER) positivity (ER(-) vs. ER(+) p = 0.046). SABT1 expression was also significantly correlated with downstream regulated genes IL-4 and MAF-1 (Pearson's correlation coefficient r = 0.21 and r = 0.162) and SATB2 (r = 0.506). After a median follow up of 10 years, there was a trend for higher SATB1 expression to be associated with shorter overall survival (OS). Higher levels of SATB2 were also found in malignant compared to background tissue (p = 0.049). SATB2 expression increased with increasing tumour grade (grade 1 vs. grade 3 p = 0.035). SATB2 was associated with ER positivity (ER(-) vs. ER(+) p = 0.0283) within ductal carcinomas. Higher transcript levels showed a significant association with poorer OS (p = 0.0433).

**Conclusion:**

SATB1 mRNA expression is significantly associated with poor prognostic parameters in breast cancer, including increasing tumour grade, TNM stage and NPI. SATB2 mRNA expression is significantly associated with increasing tumour grade and poorer OS. These results are consistent with the notion that SATB1 acts as a 'master genome organizer' in human breast carcinogenesis.

## Introduction & Background

Our understanding of the mechanisms involved in gene expression has so far exceeded our appreciation of their complex and subtle regulation. Tissue specific transcription is controlled by transcription factors, binding to enhancers or promoters, activating or suppressing expression of genes. In addition to this, the packaging of DNA into chromatin and its subsequent organization has significant implications for the regulation of gene activity [1]. The function of transcription factors therefore includes both stimulation of RNA polymerase via interactions with mediator complexes and regulation of chromatin accessibility via recruitment of histone-modifying enzymes or nucleosome-remodelling complexes [2,3]. Recent studies have demonstrated the importance of higher-order chromatin architecture in the regulation of gene expression [4,5]. The fundamental role of chromatin remodelling in coordinating expression of multiple genes has also been established [6-9]. Nuclear matrix attachment regions (MARs) are AT-rich DNA sequences involved with higher-order chromatin organization, long-range enhancer function, extension of chromatin modifications and dynamic tethering of chromatin loops [10-17]. MARs have been implicated in the regulation of gene expression due to their proximity to transcriptionally active DNA [18-23]. MARs form specific binding sites on the minor grove of the AT-rich DNA for proteins that recognize structure, rather than specific sequence. Furthermore, binding is thought to be primarily to the sugar-phosphate backbone without direct contact with specific bases [24]. Hence, unlike classic transcription factors which target individual genes, AT-rich DNA binding proteins can have many potential binding sites on multiple genes [25-28]. Several proteins have been identified that bind to MARs, including the Special AT-rich Sequence-Binding protein 1 (SATB1) [24].

SATB1 acts as a cell-type specific genome organizer regulating gene expression and coordinating cellular delineation in the haematopoietic lineage [29-31]. SATB1 is critical for differentiation and is expressed at a high levels in thymocytes [24], whereas expression is down-regulated in mature T cells [20,29-33]. SATB1 ablation has been associated with disregulation affecting multiple genes in T-cells [20,32]. Critical genes involved in cell proliferation such as c-myc are also directly regulated by SATB1, and in SATB1-null thymocytes, c-myc cannot be induced in response to mitogen stimulation [32]. Whilst SATB1 can function as a transcriptional activator [30], repressor activity has been demonstrated for certain genes [32,34]. Involvement of SATB1 in other developmental and differentiation pathways has also been demonstrated [35], including the control of neuronal differentiation within the developing CNS [36,37].

In addition to its emerging physiological role, SATB1 expression has recently been found to contribute to breast cancer growth and metastasis. Han et al. [38] found that of 24 breast epithelial cell lines examined, SATB1 mRNA and protein expression was limited to those with metastatic phenotype. RNA-interference-mediated knockdown of SATB1 in aggressive cancer cells altered the expression of >1,000 genes, effectively reversing tumourigenesis by restoring breast-like acinar polarity, anchorage-dependent growth, inhibition of cellular proliferation and invasive capacity in vitro; and tumour growth and metastasis in vivo. On the contrary, SATB1 expression in non-aggressive cells induced the malignant phenotype and in vivo metastatic competence. The authors demonstrate that SATB1 orchestrates epigenetic modifications at target loci, up-regulating metastasis associated genes and down-regulating tumour suppressors. Expression in 28 primary breast tumours was found to be greatest in all poorly differentiated infiltrating ductal carcinomas with only low-level expression in some moderately differentiated tumours and complete absence in adjacent normal tissue [38]. Nuclear staining for SATB1 was also found to be of prognostic significance in a cohort of 1,318 breast cancer cases. Higher SATB1 expression levels were associated with shorter overall survival (OS) times using Kaplan-Meier analysis of 985 ductal carcinomas. Multivariate analysis confirmed SATB1 to be an independent prognostic factor for breast cancer [38]. Zheng et al. [39] have also found SATB1 expression in aggressive rather than non-aggressive breast cancer cells. Similarly, the introduction of SATB1 into non-metastatic cells led to the induction of invasive tumors in mice; whereas SATB1 silencing returned cells to their normal phenotype and prevented metastasis and tumor growth. The research implicates SATB1 as a master regulator of metastatic competence in breast cancer.

SATB2 is a close homologue of SATB1, recently associated with cleft palate and other craniofacial dysmorphologies [40-42]. SATB2 is also a MAR-binding protein, encoding a cell type-specific transcription factor involved in the transcriptional regulation of large chromatin domains. It has been demonstrated to modulate immunoglobulin μ gene expression in pre-B cells [10]. SATB2 has also been implicated as a developmental regulator of neuronal differentiation. In contrast to SATB1, SATB2 expression has been identified in both malignant and non-malignant cell lines [38].

In this study, the expression profile of SATB1 and SATB2 is assessed in a cohort of women with breast cancer. In addition the levels of downstream genes including Interleukin 4 (IL-4), Interleukin 13 (IL-13) and MAF1 were determined. Transcript levels were evaluated against established pathological parameters and clinical outcome over a 10 year follow-up period.

## Materials and methods

### Patients and samples

Institutional guidelines, including ethical approval and informed consent, were followed. Breast cancer tissues (n = 115) and normal background tissues (n = 31) were collected immediately after excision during surgery and stored at -80°C until use. A consultant pathologist examined haematoxylin and eosin stained frozen sections to verify the presence of tumour cells in the collected samples. Normal tissue was derived from the background breast parenchyma of breast cancer patients within the study group. Medical notes and histology reports were used to extract the clinico-pathological data (Table 1). A customized database was established to record the data.

**Table 1:** Clinical and pathological data.

**Parameter**	**Category**	**Number**
**Node status**	Node positive	54
	Node negative	73
		
**Tumour grade**	1	24
	2	43
	3	58
		
**Tumour type**	Ductal	98
	Lobular	14
	Medullary	2
	Tubular	2
	Mucinous	4
	Others	7
		
**TNM staging**	1	70
	2	40
	3	7
	4	4
		
**Outcome**	Disease free	90
	Alive with metastasis	7
	With local recurrence	5
	Died of breast cancer	16
	Died of unrelated disease	9

Note: missing values reflect discarded/uninterpretable values.

### Materials

RNA extraction kits and reverse transcription kits were obtained from Sigma-Aldrich Ltd (Poole, Dorset, England, UK). The PCR primers were designed using Beacon Designer (Palo Alto, CA, USA) and synthesized by Sigma-Aldrich. Custom made hot-start Master mix for quantitative PCR was obtained from Abgene (Surrey, England, UK) [43,44].

### Tissue processing, RNA extraction and cDNA synthesis

Frozen sections of tissue were cut at a thickness of 5–10 mm and kept for routine histological analysis. An additional 15–20 sections were mixed and homogenized using a hand-held homogenizer in ice-cold RNA extraction solution. The concentration of RNA was determined using UV spectrophotometry. Reverse transcription was carried out using a reverse transcription kit with an anchored olig (dT) primer supplied by Abgene, using 1 mg of total RNA in a 96-well plate. The quality of cDNA was verified using β-actin primers (Table 2).

**Table 2:** 

**Primers for SATB1**	
AGGAAAACCGACAGAAGAC	SATB1-F
ACTGAACCTGACCGTACACCCACGTCTTGTATGAAACT	SATB1-Zr
**Primers for SATB2**	
AGGAGTTTGGGAGATGGTAT	SATB2-F
ACTGAACCTGACCGTACACCCAGAACACAATAGTCTGAA	SATB2-Zr
**Primers for IL-4**	
CACCTTACAGGAGATCATCAA	IL4-F
ACTGAACCTGACCGTACAAGCAAAGATGTCTGTTACGG	IL4-Zr
**Primers for IL-13**	
ACTCTGTTCTTGGAAACCTG	IL13-F
ACTGAACCTGACCGTACACACACTGTAATGCATGATCC	IL13-Zr
**Primers for MAF-1**	
ATTCTGGAGAGCGAGAAGT	maf-F
ACTGAACCTGACCGTACAGCTTCTCGTATTTCTCCTTG	maf-Zr
**Primers forί-actin**	
ATGATATCGCCGCGCTCGTC	
CGCTCGGTGAGGATCTTCA	

### Quantitative analysis

The level of SATB1 and SATB2 transcripts from the above prepared DNA were determined using real-time quantitative PCR based on the Amplifluor technology, modified from a method reported previously [43,44]. In addition the levels of IL-4, IL-13 and MAF1 were determined. The PCR primers were designed using Beacon Designer software, but to the reverse primer an additional sequence, known as the Z sequence (5'-actgaacctgaccgtaca-3') which is complementary to the universal Z probe (Intergen Inc., Oxford, UK) was added. The product expands one intron. The primers used are detailed in Table 2. The reaction was carried out using Hotstart Q-master mix (Abgene), 10 pmol of specific forward primer, 1 pmol reverse primer which had the Z sequence, 10 pmol of FAM (fluorogenic reporter dye, carboxyfluorescein) tagged probe (Intergen Inc.), and cDNA from 50 ng of RNA. The reaction was carried out using the IcyclerIQ (Bio-Rad Ltd, Hemel Hempstead, England, UK), which is equipped with an optic unit that allows real-time detection of 96 reactions, under the following conditions: 94°C for 12 min and 50 cycles of 94°C for 15 sec, 55°C for 40 sec, and 72°C for 20 sec. The levels of the transcript were generated from a standard that was simultaneously amplified with the samples. The levels of gene expression were then normalized against the housekeeping gene β-actin, which was already quantified in these specimens, to correct for varying amounts of epithelial tissue between samples [45]. The primers used for β-actin are detailed in Table 2. With every PCR run, a negative control without a template and a known cDNA reference sample as a positive control, were included.

### Statistical analysis

The Mann-Whitney *U*-test (comparison of median copy number) and two-sample *t*-test (comparison of mean copy number) were used for statistical analysis of absolute and normalised gene copy number. The transcript levels within the breast cancer specimens were compared to normal background tissues and analyzed against conventional pathological parameters and clinical outcome over a 10 year follow-up period. Within the tumour samples, the correlation between SABT1 and downstream regulated genes was examined using Pearson's correlation coefficient. In each case the true copy number was used for statistical analysis and hence the samples were not classified as positive or negative. The statistical analysis was carried out using Minitab version 14.1 (Minitab Ltd. Coventry, England, U.K.) using a custom written macro (Stat 2005.mtw). For purposes of the Kaplan-Meier survival analysis, the samples were divided arbitrarily into two groups, 'high transcript level' or 'low transcript level', for each gene. The cut-off was guided by the Nottingham Prognostic Index (NPI) value, with which the value of the moderate prognostic group was used as the dividing line at the start of the test. Survival analysis was performed using SPSS version 12.0.1 (SPSS Inc. Chicago, IL, USA).

## Results

### SATB1

The SATB1 expression profiles were determined both in absolute terms and normalised against β-actin in order to correct for varying amounts of epithelial tissue between samples (Table 3). SATB1 was found to be expressed in both normal/benign breast tissue and breast cancer specimens. Significantly higher levels were found in the breast cancer specimens compared to the background tissue (absolute median copy number 24.49 vs. 7.02, p = 0.0167, normalized mean copy number 0.74 vs. 0.408, p = 0.037). The expression of SATB1 mRNA was demonstrated to increase with increasing Nottingham Prognostic Index (NPI), NPI-1 vs. NPI-3 (absolute median copy number 9.0 vs. 39.9, p = 0.0614); NPI-2 vs. NPI-3 (normalized median copy number 0.095 vs. 0.432, p = 0.0495). The expression of SATB1 mRNA was also demonstrated to increase with increasing TNM stage, TNM-1 vs. TNM-2 (absolute median copy number 15.0 vs. 53.1, p = 0.0264, absolute mean copy number 65 vs. 148, p = 0.035) and increasing tumour grade, grade 1 vs. grade 3 (absolute mean copy number 45.6 vs. 111, p = 0.017, normalized mean copy number 0.347 vs. 0.89, p = 0.038); grade 2 vs. grade 3 (absolute median copy number 6.53 vs. 37.32, p = 0.0437); grade 1 vs. grade 2 and 3 (absolute mean copy number 45.6 vs. 94, p = 0.021). In addition, a significant association with ductal type was found, ductal vs. mucinous (absolute mean copy number 95 vs. 20.9, p = 0.0001, normalized mean copy number 0.77 vs. 0.331, p = 0.017). Transcript levels were also significantly associated with oestrogen receptor (ER) positivity, ER(-) vs. ER(+) (normalized mean copy number 0.94 vs. 0.456, p = 0.046). Within the tumour samples, we examined the correlation between SABT1 and the downstream regulated genes: IL-4, IL-13 and MAF-1 using Pearson's correlation coefficient. We observed a significant correlation between SATB1 and IL-4 (r = 0.21), MAF-1 (r = 0.162) and SATB2 (r = 0.506). There was no correlation with IL-13 (r = 0.026).

**Table 3:** Summary of expression profiles for the overall cohort, followed by subgroup analysis for tumour specimens and benign specimens.

	**Overall**	**Tumour**	**Benign**
**SATB1**			
Absolute	78.7 (0–976.1, 15.0)	88.6 (0–912.9, 24.5)	51.1 (0–976.1, 7.0)
Normalised	0.80 (0–23.49, 0.19)	0.74 (0–8.26, 0.20)	0.41 (0–1.49, 0.22)
			
**SATB2**			
Absolute	48.1 (0–2104.2, 0.0)	59.9(2104.2, 0.1)	9.84 (0–213.07, 0.0)
Normalised	0.12 (0–2.09, 0)	0.14 (0–2.09, 0)	0.05 (0–0.54, 0)

Values represent the true copy number of mRNA transcripts (absolute and normalised against ί-actin) and are expressed as mean (range, median).

**Figure 1:**
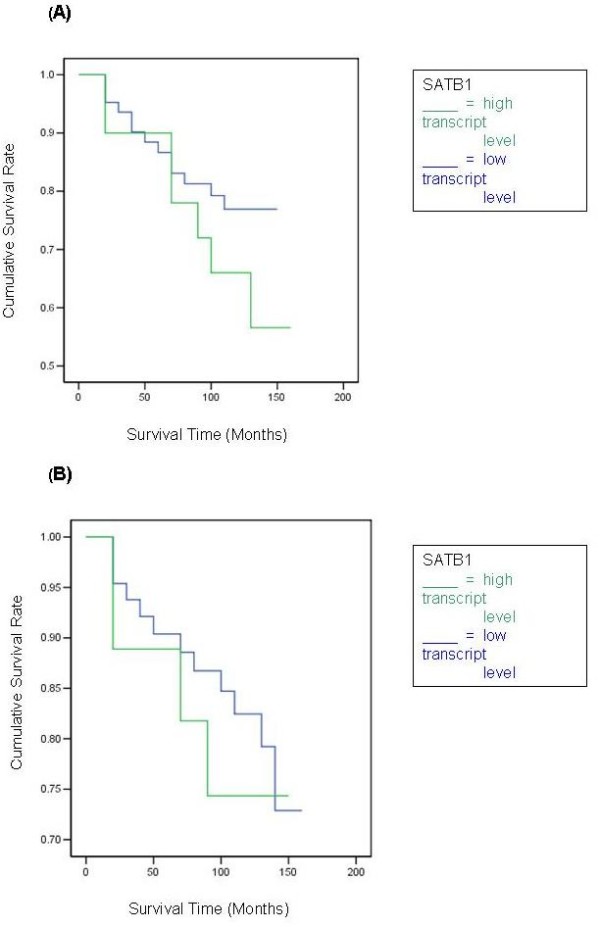
**Kaplan Meier survival curves for SATB1**. Survival times are expressed as mean (95% Confidence Interval) months. **a) SATB1 Disease Free Survival **High level: 133.8 (122.6–145.0), Low level: 107.6 (84.6–130.7), *p *= 0.1070. **b) SATB1 Overall Survival **High level: 136.0 (125.3–146.6), Low level: 121.3 (99.7–143.0), *p *= 0.4793.

After a median follow up of 10 years, there was a trend for tumours with higher SATB1 expression levels to be associated with shorter OS times, although this did not reach statistical significance. The disease free survival (DFS) and OS curves for women with tumours which were classified as having 'high levels' of SATBI transcript was not found to differ significantly from that of their 'low level' counterparts, Figure 1a,b. The survival curves show higher levels of SATB1 were of marginal benefit in predicting lower DFS (p = 0.174, NS).

### SATB2

The SATB2 expression profiles were also determined both in absolute terms and normalised against β-actin (Table 3). SATB2 was found to be expressed in both normal/benign breast tissue and breast cancer specimens. Significantly higher levels were found in the breast cancer specimens compared to the background tissue (absolute mean copy number 60 vs. 9.8, p = 0.065, normalized mean copy number 0.138 vs. 0.053, p = 0.049). The expression of SATB2 mRNA was also demonstrated to increase with increasing tumour grade, grade 1 vs. grade 3 (normalized mean copy number 0.0380 vs. 0.169, p = 0.035). In addition, a significant association with ductal type was found, ductal vs. mucinous (absolute mean copy number 64 vs. 2.56, p = 0.061, normalized mean copy number 0.158 vs. 0.004, p = 0.0007); ductal vs. medullary (absolute mean copy number 64 vs. 0.0135, p = 0.050); ductal vs. others (normalized mean copy number 0.158 vs. 0.045, p = 0.032). Within the ductal carcinoma subgroup, transcript levels were also significantly associated with ER positivity, ER(-) vs. ER(+) (absolute median copy number 0.0 vs. 10.0, p = 0.0283).

**Figure 2:**
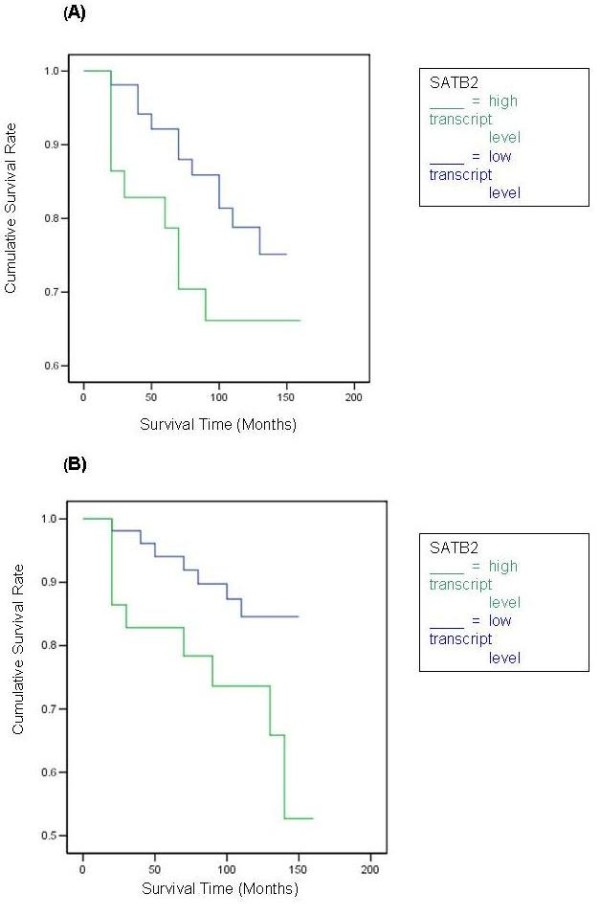
**Kaplan Meier survival curves for SATB2**. Survival times are expressed as mean (95% Confidence Interval) months. **a) SATB2 Disease Free Survival **High level: 128.2 (118.3–138.2), Low level: 117.2 (95.9–138.5), *p *= 0.1001. **b) SATB2 Overall Survival **High level: 133.7 (124.9–142.6), Low level: 120.0 (100.0–140.2), *p *= 0.0433.

The DFS curve for women with tumours which were classified as having 'high levels' of SATB2 transcript was not found to differ significantly from that of their 'low level' counterparts, Figure 2a. Only a marginal benefit in predicting DFS was identified. However, tumours classified as having higher transcript levels showed a statistically significant association with a poorer OS (Figure 2b, p = 0.0433).

## Discussion

In the study by Han et al. [38] SATB1 expression was found to be greatest in all poorly differentiated infiltrating ductal carcinomas with only low-level expression in some moderately differentiated tumours and complete absence in adjacent normal tissue. Expression was not found to be restricted to late stage disease, but already present in a subset of early primary tumours without lymph node metastasis. SATB1 was found to be of high prognostic significance, independent of the lymph node status and nuclear staining was associated with shorter OS [38]. In keeping with these results, we also found significantly higher levels of SATB1 mRNA in the breast cancer specimens compared to the background tissue and significant association with poor prognostic parameters including, increasing tumour grade, TNM stage and NPI. Although we observed a trend for tumours with higher SATB1 mRNA expression levels to be associated with shorter OS times, this failed to reach statistical significance. Similarly, significantly higher levels of SATB2 mRNA were found in the breast cancer specimens compared to the background tissue and expression was significantly associated with increasing tumour grade. In contrast to SATB1, tumours classified as having higher transcript levels of SATB2 were significantly associated with a poorer OS.

In-vitro and in-vivo studies have demonstrated that SATB1 can induce the malignant and metastatic phenotype in breast cells, through widespread alteration of gene expression profiles [38]. In particular, targeted up-regulation has been demonstrated in poor prognosis genes associated with proliferation, metastasis, angiogenesis, degradation of the extra-cellular matrix and tumour invasion, such as metastasin (S100A4), VEGFB, matrix metalloproteases 2, 3 and 9, TGFB1, endothelial growth factor receptor and epidermal growth factor subfamily members, including HER-2/NEU. Furthermore, specific down-regulation of several tumour suppressor genes including BRMS1, KAI1, NME1, and KISS1 has been reported [38]. In our study, correlation between SABT1 mRNA expression and the downstream regulated genes IL-4 and MAF-1 was identified. IL-4 has recently been implicated in the resistance to cell death observed in epithelial tumours, including breast cancer. IL-4 appears to function as an autocrine survival factor by amplifying expression of anti-apoptotic proteins and preventing therapy related cell death. IL-4 blockade has been shown to decrease growth rate and sensitize cells to chemotherapy [46]. MAF-1 has been demonstrated to be a key regulator of RNA polymerase (pol) III which produces essential components of the biosynthetic machinery and is therefore a key determinant of cell growth and proliferation [47,48]. MAF-1 has also been implicated in regulation of the transformation state of cells [49]. Hence, expression of SATB1 could represent a singular event with profound implications for tumourigenesis and metastasis in human breast cancer. This would be consistent with the emerging interplay between epigenetics, chromatin remodelling and cancer [50-52]. Tumourigenesis and metastasis represent key stages in the development and progression of human cancer. Both are multi-step processes where genetic alterations are associated with characteristic changes in phenotype. Acquisition of metastatic competence is the prelude to widespread dissemination, resulting in the conversion of a local pathology into a systemic disease with associated mortality. [53,54]. How cells are initiated along a pathway to malignancy and how malignant cells gain metastatic potential is not well understood. Conventionally, cells are believed to evolve through sequential and additive genetic changes resulting in 'gain of function' and metastatic capacity. However, breast carcinomas with known clinical outcomes have recently been associated with characteristic genetic profiles [55-58]. Hence, some cells within the primary tumour may already have the requisite gene expression pattern. The contemporary viewpoint is that such expression profiles may be simultaneously acquired through specific master changes in global genomic organization [38]. In keeping with this, SATB1 can bind to numerous genomic sites, influencing chromatin organization and orchestrating transcription of multiple genes.

The functional relevance of genomic organization, particularly towards transcription, requires the reconciliation of two distinct paradigms, one suggesting that genomic organization merely reflects nuclear processes including transcription, and the other proposing that dynamic genome organization plays a decisive role in its function [59]. SATB1 represents a novel class of transcription regulator and has been frequently referred to as a genome organiser. SATB1 appears to serve a dual purpose, structurally as a component of chromatin architecture and functionally as a transcription factor [38]. Chromatin remodelling is emerging as an important effector mechanism of epigenetic regulation. The role of SATB1 is therefore instrumental to the relationship between higher order chromatin organization and global transcriptional regulation [60]. SATB1 is a MAR binding protein involved in targeting chromatin remodelling in a tissue-specific manner at specific transcriptionally active chromosomal sites. It interacts with AT-rich sequence motifs within core unwinding elements that become unpaired under conditions of torsional/superhelical stress. Such base-unpairing regions (BURs) augment the potential for enhancers to act over large distances [10,24,28,36,37,61-64]. The protein selectively tethers multiple genomic loci and is localized to characteristic 'cage-like' three dimensional protein scaffold that anchors loops of chromatin, forming a dynamic chromatin 'loopscape' [20,30,60,62]. By acting as a 'docking site' it recruits chromatin remodelling complexes and regulates histone modifications and nucleosome positioning over long stretches of DNA [20,30,65,66].

The regulation of the activity of MAR-binding proteins remains poorly understood. Post-translational modification of SATB1 may provide a subtle refining mechanism for regulation of chromatin architecture and gene transcription [60]. In this way, SATB1 may act as repressor or activator depending upon the physiological context [18,19,30,67-69]. At an early stage of apoptosis, SATB1 is irreversibly inactivated by caspase 6 cleavage, resulting in rapid dissociation from chromatin and MARs [70]. SATB1 is also one of the few nuclear proteins harbouring the PDZ protein-protein interaction domain, permitting interaction with many nuclear proteins [20]. Intriguingly, SATB1 does not contain a classical nuclear localization signal and a novel N-terminus sequence motif has been found to mediate nuclear localization [29]. In contrast to SATB1, SATB2 can be reversibly and dynamically modified by SUMOylation (covalent conjugation of the small ubiquitin-related modifier), which appears to modulate its activity as a transcription factor. SUMOylation is also involved in targeting SATB2 to the nuclear periphery [10,71,72]. The structural similarity between SATB1 and SATB2 implies analogous roles in transcriptional regulation. Furthermore, these proteins are highly conserved across species [36,37,40,73]. Further studies are required to determine the genome-wide targets of SATB1 and the multitude of interacting factors recruited. Furthermore, the process underlying tissue specificity of regulated genes and the mechanisms of transcriptional up- or down-regulation will need to be fully elucidated to optimise the impact of any potential therapeutic manipulation strategies [74]. Chromatin organizers are unlikely to be unique to breast cancer pathogenesis and study of other human cancers is warranted.

Limitations of the present study included the use of background parenchyma from breast cancer patients to provide 'normal tissue' for comparison. Ideally, such material should be derived from patients without breast cancer in order to avoid any 'field change' which may exist within cancer bearing tissues. Although the sample size and follow-up period were substantial, it is possible that a larger cohort may have influenced several results which approached, but failed to reach, statistical significance. Finally, in addition to the measurement of mRNA transcript levels, quantitative analysis of protein expression should be undertaken to ensure concordance.

## Conclusion

SATB1 mRNA expression is significantly associated with poor prognostic parameters in breast cancer, including increasing tumour grade, TNM stage and NPI. SATB2 mRNA expression is significantly associated with increasing tumour grade and poorer overall survival. These results are consistent with the notion that SATB1 acts as a 'master genome organizer' in human breast carcinogenesis. Further studies are required to elucidate their contribution the development and progression of the malignant phenotype.

## Conflicts of interests

The authors declare that they have no competing interests.

## Authors' contributions

NP – literature review, data analysis and interpretation, manuscript preparation, manuscript editing. WJ – primer design, RNA extraction, reverse transcription, quantitative PCR, statistical analysis. RM – study design and planning. RN-study design and planning. KM – study design, patient recruitment, statistical analysis, data interpretation, manuscript editing. All authors read and approved the final manuscript.
